# LEADERS AND CONSULTANTS FROM AN AGING CENTER IN MICHIGAN: PRODUCTIVE AND TAILORED ENGAGEMENT WITH AFRICAN AMERICANS

**DOI:** 10.1093/geroni/igae098.1297

**Published:** 2024-12-31

**Authors:** Tam Perry, Wilma Stringer, Cynthia Howell, Vanessa Rorai, Amanda Horn, Jamie Mitchell, Kent Key

**Affiliations:** Wayne State University, Detroit, Michigan, United States; Healthier Black Elders Center, Detroit, Michigan, United States; Flint Healthier Black Elders Center, Flint, Michigan, United States; Healthier Black Elders Center, Detroit, Michigan, United States; Detroit Healthier Black Elders Center, Southgate, Michigan, United States; University of Michigan, Ann Arbor, Michigan, United States; Michigan State University East Lansing, East Lansing, Michigan, United States

## Abstract

The Healthier Black Elders Center (HBEC) is the community outreach core for the Michigan Center for Urban African American Aging Research. Established in 1997, this center offers health education to older adults in Detroit and expanded to Flint, Michigan in 2021 and maintains a research registry of over 1100 older Detroiters and over 200 older Flint residents. HBEC Detroit launched a consulting program in 2020 with the Community Advisory Board (CAB) members to offer personal and professional perspectives regarding research. Through this program, CAB members have 1) disseminated knowledge about community engaged research approaches 2) offered feedback on comprehensibility and ease of use to individual researchers on instrument content and research design as well as 3) offered insights beyond the academy (technology and philanthropic sectors). This program has created opportunities for older Black adults to provide direct feedback on product, funding initiatives and research design. This presentation will provide an overview of the initiatives, recruitment, and retention strategies of a long-established center. This presentation will showcase the Consulting program as a way to engage leaders in its center’s work as well as provide learning and growth for its Detroit members. This presentation will also highlight the process of tailoring, adapting, and launching the HBEC program for the Flint community.

